# The Gymnasium at the Virchow Krankenhaus

**Published:** 1913-09-20

**Authors:** A. Laqueur

**Affiliations:** Berlin, Director of the Gymnasium and the Hydrotherapeutic Institution.


### THE GYMNASIUM AT THE VIRCHOW KRANKENHAUS.

By Dr. A. LAQUEUR, Berlin, Director of the Gymnasium and the Hydrotherapeutic Institution.

The Gymnasium at the Virchow Krankenhaus is a hall, 26 metres in length and 11 metres in width, which receives its light from three sides. The hall is divided into three sections by rows of columns. The middle section is the biggest and contains the apparatus properly used for the medico-mechanical treatment; one of the smaller sections contains the apparatus for the treatment of scoliosis, and the other the arrangements for the exercises of those invalids who suffer from tabes. The medico-mechanical apparatus is constructed for the greater part after the system of Zander (Stockholm), but the workmanship is German, and presents small but unimportant deviations from the original Zander system. Most of the Zander apparatus (there are twenty-four appliances in the whole) is put into motion by the patients themselves. The apparatus may be used for active movements and at the same time for passive movements of the antagonistical muscles.

At one of the side walls of the great middle section the passive apparatus is placed, which is put into motion by a motor with a common axis. The middle part contains, besides the Zander apparatus, a rowing apparatus, a veloriped, and a chair for the treatment of asthma by the way of mechanical compression of the thorax during the expiration (system of Dr. Boghean). Also the apparatus for the treatment of scoliosis are on the whole constructed after the system of Zander. The apparatus for the treatment of tabes follow the statements of Frenkel and Goldscheider. The total amount for the whole furniture of the Gymnasium came to 25,000 M., of which about 18,000 M. fall on the Zander apparatus.

In the Gymnasium those patients are treated who have been sent there from different divisions of the hospital. Between fifty and sixty patients are treated there daily, the total number of treatments amounting in the year 1912 to 1,598. Especially those patients are treated here who have suffered a stiffening and disorder of function of their joints through fractures, luxations, distortions, and so on. With these, as with most other patients treated here, the mechanical-therapeutical treatment is combined with kneading and manual mobilisation. Further on, many patients with disturbance of movement caused by chronic arthritis deformans, gonorrhœal arthritis, and so on, are treated here. In these cases the treatment contributes essentially to restore the mobility, but usually it is preceded by a few weeks' treatment (of the patients) with baths and kneading so that they may lose the painfulness of their joints. We also treat the patient with light exercises after acute rheumatism of the joints to fight against the feeling of weakness which often remains after this illness.

Besides, we had good results by internal illness as gout, diabetes, and adipositis, as the exercises incite and heighten the metabolism. We often had good results with cautious exercises with patients suffering from chlorosis, as well as with neurasthenic patients to fight against the feeling of weakness. Frequently we apply mechanical-therapeutical exercises of respiration for the after-treatment and removal of pleuritic exudations or pleuritic adhesions, also for the after-treatment of pneumonia. Also to some forms of chronic suffering from gall-stones and cholecystitis our systematical exercises for breathing had good results in the advancement of the circulation in the liver. Further, we had excellent results with the treatment of the above-mentioned breathing-chair of Dr. Boghean in the case of those patients suffering from bronchial asthma and chronic emphysema.

And, lastly, we also use the active and passive apparatus of Zander for palsy, as well central as peripheral ones, especially for spastic palsy. For ataxia, in consequence of tabes, we often could gain good results with systematical exercises by apparatus only for this kind of treatment, viz., the deliberate exercise of walking; in many cases even a great amelioration was stated.

Arrangements for hydrotherapy and electrotherapy may be found in the same building, which are also under the guidance of the Director of the Gymnasium. Most patients who are treated in the Gymnasium also stand under treatment in the other division. Besides the Director, an assistant doctor is busy there. The rest of the employees of this institution consist of an upper nurse, a special keeper for the Gymnasium, two keepers for the bathing house, who are at the same time learned kneading men, two female keepers of the bathing house, and kneading women, as well as different other helps.

